# Mitochondrial genome assembly and phylogenetic analysis of *Upeneus japonicus* (Houttuyn, 1782) from the East China Sea

**DOI:** 10.1080/23802359.2022.2095939

**Published:** 2022-07-18

**Authors:** Li Wang, Shouqiang Wang, Ye Lin, Shenghao Liu, Panjiao Liang, Linlin Zhao

**Affiliations:** a School of Municipal and Environmental Engineering, Shenyang Jianzhu University, Shenyang, China; b Key Laboratory of Marine Eco-Environmental Science and Technology, First Institute of Oceanography, Ministry of Natural Resources, Qingdao, China

**Keywords:** *Upeneus japonicus*, mitochondrial genome, phylogenetic analysis

## Abstract

The mitochondrial genome of *Upeneus japonicus* was successfully assembled by high-throughput sequencing data in this study. This is the first report on the complete mitochondrial genome of *U. japonicus*, with a total length of 16,535 bp, including 13 protein-coding genes (PCGs), two ribosomal RNA genes (rRNAs), 22 transfer RNA genes (tRNAs), and a control region (D-loop). The overall base composition is 26.05% A, 26.10% T, 29.14% C, and 18.71% G. Phylogenetic analysis showed that *U. japonicus* was grouped with its sister species *U. tragula*. The mitochondrial complete genome study of *U. japonicus* would lay the foundation for further studies in population genetics and evolutionary analysis.

*Upeneus japonicus* belongs to the family Mullidae, which is widely distributed in the Western Pacific, such as the Philippines, South Korea, the South China Sea, the East China Sea, the Yellow Sea, and the Bohai Sea (Randall et al. 1993; Markevich and Balanov 2012). It is the bottom-dwelling fish with a habitat depth of about 4–90 m (Uiblein and Gledhill 2015). There is often a high degree of similarity among morphological characteristics of species in *Upeneus*; therefore, it calls for the development of molecular markers to distinguish them (Uiblein and Gledhill 2015). In this study, we analyzed the complete mitochondrial genome of *U. japonicus*, determined its phylogenetic position, and provided genetic information for further biological and phylogenetic studies.

The samples were captured in the East China Sea (27°21′N, 122°6′E) in October 2021 by Agassiz trawl, and stored at First Institute of Oceanography, Ministry of Natural Resources (http://en.fio.org.cn/, no. FIORBF02, Linlin Zhao, zhaolinlin@fio.org.cn). A small piece of muscle near the dorsal fin was taken, placed in absolute ethanol, and stored at −80 °C. Total genomic DNA was extracted using the DNeasy Blood & Tissue Kit (QIAGEN, Hilden, Germany). Paired-end sequencing of total genomic DNA is based on the high-throughput MGISEQ-T7 platform with an insert size of 350 bp. Mitochondrial genome assembly was performed on clean data using MitoFinder v1.4.1 (Allio et al. 2020) based on the reference sequence of *U. tragula* (GenBank accession number OK236377). Then, MitoFish was used to annotate the assembled mitochondrial genome (Iwasaki et al. 2013). The mitochondrial genome data have been uploaded to the National Center for Biotechnology Information GenBank (NCBI) database with accession number ON116346.

The total length of the mitochondrial genome of *U. japonicus* is 16,535 bp, including 13 protein-coding genes (PCGs), two ribosomal RNA genes (rRNAs), 22 transfer RNA genes (tRNAs), and a control region (D-loop). The overall base composition is 26.05% A, 26.10% T, 29.14% C, and 18.71% G. The two rRNAs are 12S rRNA with length of 949 bp and 16S rRNA with length of 1669 bp. The 22 tRNAs range in length from 66 bp to 74 bp. Among the 13 PCGs, except for CO1 whose start codon is GTG, the others use ATG as the start codon. Among them, Cytb is terminated by a truncated stop codon T. ND2 and ND4 are terminated by AGA. ATP8, ND3, and ND6 with a TAG stop codon, the remaining seven PCGs (ND1, CO1, CO2, ATP6, CO3, ND4L, and ND5) are all terminated by TAA. ND6 and eight tRNAs (tRNA-Glu, tRNA-Pro, tRNA-Gln, tRNA-Ala, tRNA-Asn, tRNA-Cys, tRNA-Tyr, and tRNA-Ser) are on the light chain, the other genes are on the heavy chain.

The phylogenetic tree was conducted using MEGA7.0 based on the nucleotide sequences of 13 PCGs from 14 species of the order Perciformes and an outgroup species of the order Pleuronectiformes (Kumar et al. 2016). Phylogenetic analysis showed that *U. japonicus* possessed the closest relationship with *U. tragula*. The genera *Upeneus* with other three genera (*Parupeneus*, *Mulloidichthys*, *Mullus*) clustered as Mullidae, and then grouped with Callionymidae (Figure 1). This mitochondrial genome of *U. japonicus* will provide information to help understand how its relatives evolved genetically and evolutionarily.

**Figure 1.**
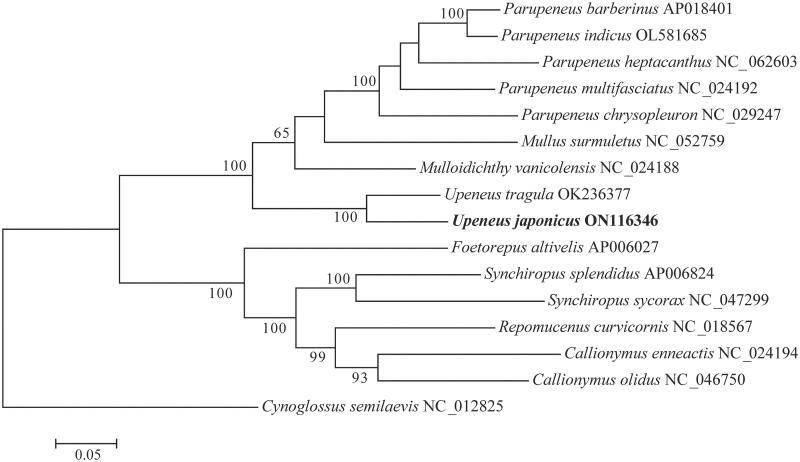
Phylogenetic tree constructed from the nucleotide sequence data of 13 protein-coding genes. The numbers on the nodes represent the credibility, and the scale bar represents the relative evolutionary distance. Values below 50% are not displayed.

## Data Availability

The genome sequence data that support the findings of this study are openly available in GenBank of NCBI at https://www.ncbi.nlm.nih.gov/ under the accession number ON116346. The sequencing data reported in this paper have been deposited in the National Genomics Data Center (NGDC, https://ngdc.cncb.ac.cn) under the BioProject number PRJCA009031, and the Bio-Sample number SAMC713164. Genome Sequence Archive (GSA) under the accession number CRR458292 (https://ngdc.cncb.ac.cn/gsa/browse/CRA006611/CRR458292).
